# Oncology Communication Skills Training: Bringing Science to the Art of Delivering Bad News

**Published:** 2015-03-01

**Authors:** Mady C. Stovall

**Affiliations:** University of Texas Health Science Center at San Antonio, San Antonio, Texas

## Abstract

Review of "Effect of communication skills training program for oncologists based on patient preferences for communication when receiving bad news: A randomized controlled trial" by Fujimori et al. (2014), Journal of Clinical Oncology, 32, 2166–2172. For a further discussion of survey research, please see the related article by Julie Ponto starting on page 168.

Few published studies reporting on controlled clinical trials in the psychosocial domain of communication techniques for delivering bad news to cancer patients exist. The landscape of oncology front-line care is dramatically changing. Nurse practitioners and physician assistants are working side by side with oncologists to care for increasing numbers of cancer survivors. When considering topics such as delivering bad news to oncology patients, it is important to base communication skills on tested theories and interventions: The need for level 1 evidence with empirical data is critical.

Considering the fact that over the course of a career, an oncologist may impart bad news an average of 20,000 times (Schmid Mast, Kindlimann, & Langewitz, 2005), front-line providers of cancer care should recognize the toll such a deed takes upon both the recipients of such dreaded news and the bearer of the news—the professional oncology care provider. The consequences of bad news delivery contribute to the lived experiences of patients as well as health-care professionals (Paul, Clinton-McHarg, Sanson-Fisher, Douglas, & Webb, 2009). Epstein and Street (2007) have reported that from a patient perspective, skilled communication by oncology providers has been associated with improved satisfaction, adherence to treatment, better health outcomes, improved recall, and better-quality understanding.

Clinician perceptions are also important, though they have been less studied. According to Baile (2011), insufficient communication training of oncology providers is a key element contributing to stress, decreased job satisfaction, and emotional burnout (Fallowfield & Jenkins, 1999; Penson, Dignan, Canellos, Picard, & Lynch, 2000).

## HISTORY OF COMMUNICATION SKILLS TRAINING

The body of research revolving around the skills needed to communicate effectively with oncology patients over the past 30 years has primarily focused on the oncology provider, specifically the oncologist (Kissane et al., 2012). Most of this research centered on provider participation training and promotion of empathetic behavior. However, as Kissane and colleagues describe (2012), the majority of communication skills training (CST) programs are based on practical advice and lack focus or consistent outcome measures across studies that could add to the literature.

In 2009, Paul and associates published a review of the literature from January 1995 to March 2009, reporting that only 41 publications of 245 (16.7%) were truly intervention studies related to breaking bad news about cancer. Only 4 of those 41 were intervention studies evaluating a patient outcome. The first consensus guidelines on breaking bad news were published in 1995 (Girgis & Sanson-Fisher), but less than 2% of studies over a 19-year period focusing on doctor-patient interaction explicitly addressed *how* providers should formulate the information in such a way as to improve patient outcomes and increase patient satisfaction (Paul et al., 2009). The field is rife with opportunities to improve established models of CST to include patient-specific outcome measures (such as adherence to therapy, health-care provider evaluations, and health-related quality-of-life scores).

## A PROVIDER-CENTERED APPROACH

A variety of patient- and provider-specific issues contribute to, and arise out of, a consultation that involves imparting bad news. Focusing on the health-care provider’s proficiency in the delivery of distressing news provides the most global approach to improving outcomes. It has been shown that an oncology provider’s lack of well-tuned communication skills diminishes patient disclosures, increases patient anxiety, and decreases satisfaction with care (Sheldon, 2005). Furthermore, an insensitive delivery approach increases distress for the recipients of bad news, may exert a lasting impact on their ability to adapt and adjust, and can lead to anger and an increased risk of litigation (Fallowfield & Jenkins, 2004).

When oncology physicians communicate well, patients are more satisfied with care, feel more in control, are more likely to follow through with treatment, are more informed, are more likely to take part in a clinical trial, and are better able to transition to palliative care (National Cancer Institute [NCI], 2014). Using an evidence-based CST approach can have a positive impact on patients and their health-care providers.

## MODELS OF COMMUNICATION SKILLS TRAINING

It is vital to examine the ways bad news is shared between providers and patients. Professional educational models now provide communication skills training, although different professions implement varied approaches (Baile et al., 2000; Edwards, Peterson, & Davies, 2006). The medical model typically uses CST relative to a particular event, such as breaking bad news, obtaining informed consent, or advanced care planning (Baile et al., 2000; Kissane et al., 2012). In the early 1990s, recognizing the importance of this topic and the level of evidence needing to be built, a consensus approach was taken to building guidelines in delivering bad news and called for detailed intervention studies (Buckman, 1992; Girgis & Sanson-Fisher, 1995). The Accreditation Council on Graduate Medical Education (ACGME) adopted a list of six core competencies in 1999, one of which is Interpersonal and Communication Skills (Swing, 2007). In 2004, standardized communication skills testing as part of the Clinical Skills Test portion of the United States Medical Licensing Examination (USMLE) Step 2 was implemented (USMLE, 2004). Physician-focused communication skills interventions aim to promote improved patient outcomes as well as improved physician satisfaction (Girgis & Sanson-Fisher, 1995; Fujimori et al., 2014).

In contrast to the medical model, undergraduate nursing education often has a focus on teaching general, transferable communication skills, such as therapeutic (or active) listening, the use of silence, or motivational interviewing (Edwards, Peterson, & Davies, 2006). More frequently than any other health professional, oncology nurses in particular participate in intimate personal exposures as part of the vulnerability experienced by individuals and families under less than optimal health conditions. Exposure to such raw infirmity (whether the malignant malady is physical, mental, spiritual, psychosocial, or a combination thereof) informs nursing practice and individual cognizance. Studies suggest that nurses spend more time with patients through the cancer care trajectory than any other member of the health-care team, and are often cited as the most trusted members of the oncology team when it comes to obtaining information (NCI, 2014).

While some advanced practice nurses in oncology may receive additional training similar to the CST training in the medical model to augment and complement the foundation of CST provided in basic nursing education, not all advanced practice programs provide this important element. *The Essentials of Doctoral Education for Advanced Nursing Practice* requires that nurses with a doctor of nursing practice (DNP) degree "develop and sustain therapeutic relationships and partnerships with patients (individual, family, or group) and other professionals to facilitate optimal care and patient outcomes" (American Association of Colleges of Nursing, 2006). With more medical and advanced practice nursing models of education recognizing the importance of this training, there is an increasing need for rigorous evidence-based practice.

Accrediting bodies from medicine and nursing are sending the message that the skills required to impart bad news are teachable. Kissane et al. (2012) published a review of the empirical psycho-oncology communication research. While there is clearly value to the research thus far, future communication studies need to address quantitative and qualitative outcomes using more effective and consistent patient and provider measures of satisfaction, understanding, and health outcomes after a bad news encounter.

## NEW EVIDENCE

Addressing the level 1 evidence gap, the most recent project by Fujimori and colleagues (2014) examines the effects of a CST program on oncologists in Japan based upon patient preferences for communication when receiving bad news. Fujimori’s team built upon their prior quantitative and qualitative surveys, showing that patient preferences for communication in a bad news situation consisted of four factors: the setting, how the news is delivered, the provision of various types of information, and emotional support (Fujimori et al., 2007a). The 2014 article by Fujimori and colleagues contributes to filling the gap in randomized controlled studies in psycho-oncology communication skills training literature, addressing both provider and patient outcomes.

Oncologists from two hospitals in Japan were invited to participate in the study. Of the 153 oncologists available in the two institutions, only 30 agreed to participate. It is interesting to note that the only significant difference in participating provider characteristics was gender: Only 11 of 153 oncologists potentially eligible for this study were women, yet of the 30 study participants, 46% were female. The patients were recruited after follow-up consultations with participating oncologists. The randomized, controlled study design aimed to identify the effects of a 2-day CST program for oncologists. The program had been previously developed using (1) patient preferences for evaluating oncologists’ behaviors during simulated consultation, (2) oncologist confidence in communicating with patients, and (3) patient levels of distress and satisfaction after the consultation. Informed consent was obtained from all study participants (oncologists and patients).

Oncologists were randomized to join an intervention group (those who attended the 2-day CST workshop) or a control group. Each group had 15 oncologists. At baseline, all oncologists participated in a consultation delivering a diagnosis of incurable advanced cancer to a simulated patient. Each oncologist completed questionnaires describing personal demographic characteristics, medical experience, and perceived self-confidence in patient communication. Recruitment of all eligible patients occurred on the day of a consultation with a participating oncologist. Each was asked to complete and return a series of questionnaires within 1 week.

Psychological distress was evaluated using the Hospital Anxiety and Depression Scale (HADS). Patient satisfaction with the oncologist’s communication during the consultation and patient trust in the oncologist were also evaluated. Demographic characteristics were also recorded. There were 267 patients providing postconsultation assessments of the 30 oncologists in the intervention group and 313 patients doing the same for the oncologists in the control group. After 1 week, a 2-day workshop was administered to the intervention group. The workshop included a didactic component, a role-play element, and discussion. The CST program was based on the four dimensions of the SHARE conceptual communication skills model (Fujimori et al., 2007a; Fujimori & Uchitomi, 2009):

- **S**upportive environment for interview
- **H**ow bad news will be delivered
- **A**dditional information the patient requests
- **RE**assurance and empathy in response to patient emotions

## STUDY MEASURES

Oncologists’ communication skills performances were measured during the simulated consultations at baseline and follow-up. The simulated patients had 3 years’ experience in medical school and had received 60 hours of training by a manual in simulating standard reactions of patients. To improve interrater reliability, the consultations were videotaped and then coded as 1 of the 27 categories analyzing provider performance in the SHARE model by 2 blinded coders who had received 30 hours of training for the coding task, independently, on 2 occasions. One week after the intervention, or 2 weeks after the initial consultation for the control group, patients were reassessed. In total, 1,192 patients were assessed at baseline and/or follow-up (response rate, 84.6%).

## STUDY OUTCOMES

A unique feature of this particular Fujimori study is the positive effect associated with participation in the CST program as shown by physician and patient outcomes. Physicians in the intervention group were rated as having significantly improved skills in expressing emotional support (*p* = .011), setting up a supportive environment (*p* = .002), and delivering information (*p* = .001), as compared to the control group. Those in the CST workshop also rated themselves higher in communication confidence (*p* = .001) than those in the control group.

Patients surveyed with HADS who had consultations with physicians in the intervention group reported being significantly less depressed (*p* = .027) than those who had interventions with physicians in the control group. Trust in their oncologist was also higher (*p* = .009) for those patients seeing the intervention group participants. Reduction in distress for the intervention group’s patients was only marginal (*p* = .05), and there was no significant difference between patients’ HADS scores relative to anxiety. Patients were equally satisfied with their oncologists’ communication style in both groups.

## STUDY LIMITATIONS

While this most recent Fujimori project is a valuable contribution to the psychosocial domain of communication literature and shows positive results for patients and health-care providers, there are many limitations to this study. One potential confounder to consider is the fact that the CST program design was specifically based upon previously reported patient preferences regarding the communication of bad news (Fujimori et al., 2007a; Fujimori et al., 2007b). The authors fully disclose this limitation and the fact that the intervention group oncologists were likely able to provide more culturally appropriate support. Tools measuring satisfaction are challenging to establish, in terms of specificity and validity. Furthermore, few questionnaires are tailored to evaluate a single visit or a single provider, and more often evaluate the entire clinical experience of a health-care experience. The authors admit that there are several limitations to the current study, and rightfully question why approximately 80% of potential participating oncologists declined to participate in the study. In addition, even though women made up almost 50% of the study physicians, female physicians represented only 7% of the 153 total available oncologists for study participation.

Recently, Gyawali, Tsukuura, Honda, Shimokata, & Ando (2015) raised interesting questions about the Fujimori study. Taking into consideration a Japanese cultural norm of behaving in a stoic manner, they suggest that Japanese physicians are potentially less likely to see value in CST. Another critique was of the recruitment of all eligible patients, not necessarily those patients who are about to get bad news.

Receiving good news about the status of any disease is surely to be better received than receiving bad news about the status of that disease. Another limitation put forth is that of the phase of disease, such as newly diagnosed or advanced disease consultation (Gyawali et al., 2015). Fujimori and his team hope that future CST studies will allow measures of both short- and long-term outcomes (2014).

## CONCLUSIONS

The field of communication skills training in oncology is still rife with opportunity to establish reliable tools of measure and interventions of potential benefit. That said, the growing body of literature increasingly demonstrates that empathetic communication skills can be learned and appears to be a method of information delivery that improves the lives of patients with cancer as well as oncology health-care providers.
